# Supplementary material for: Environmental Adaptation in the Process of Human-Land Relationship in Southeast China’s Ethnic Minority Areas and Its Significance on Sustainable Development
Source: Int J Environ Res Public Health. 2023 Feb 3;20(3):2737. doi: 10.3390/ijerph20032737 (PMC9915183; doi:10.3390/ijerph20032737)
Supplement: Supplementary file 1 [file ijerph-20-02737-s001.zip › ijerph-2084258-supplementary.pdf]

International Journal of
Environmental Research and Public Health

MDPI

Supplementary Materials

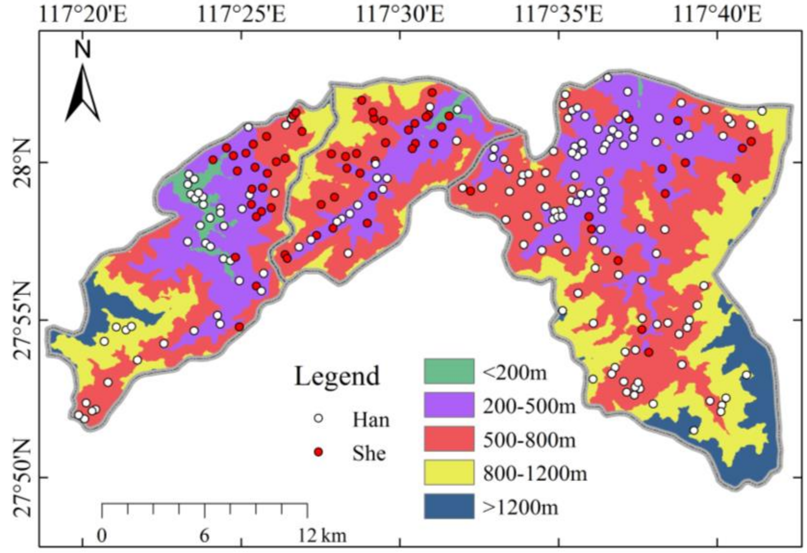

Figure S1. Human settlements in different elevations

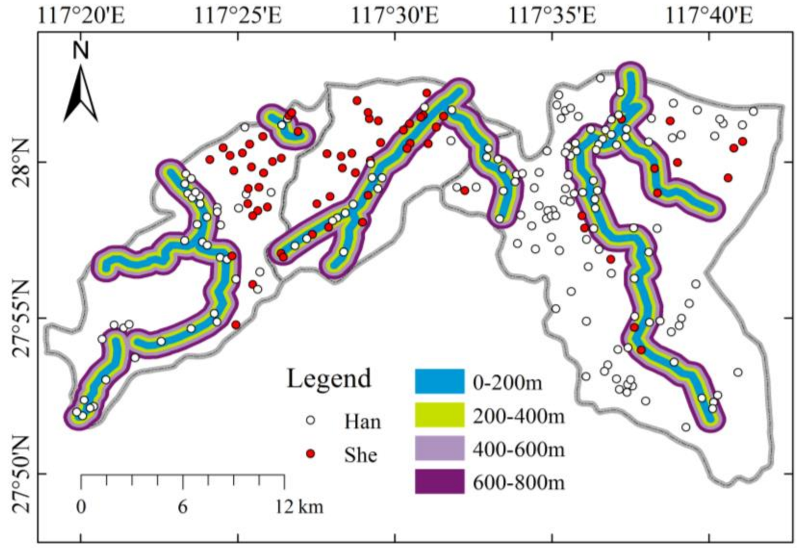

Figure S2. Human settlements in buffers of river

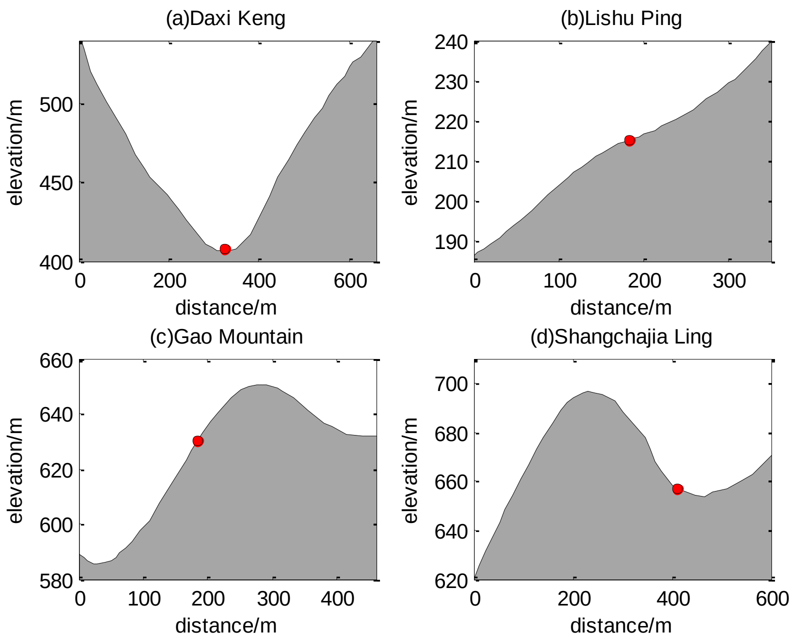

Figure S3. Topographic indication map of major place names (These toponymic points have the most obvious geomorphic features, so they are selected as representative sites for illustration.)
